# Project Khanya: results from a pilot randomized type 1 hybrid effectiveness‐implementation trial of a peer‐delivered behavioural intervention for ART adherence and substance use in HIV care in South Africa

**DOI:** 10.1002/jia2.25720

**Published:** 2021-06-24

**Authors:** Jessica F Magidson, John A Joska, Jennifer M Belus, Lena S Andersen, Kristen S Regenauer, Alexandra L Rose, Bronwyn Myers, Sybil Majokweni, Conall O’Cleirigh, Steven A Safren

**Affiliations:** ^1^ Department of Psychology University of Maryland College Park MD USA; ^2^ HIV Mental Health Research Unit Division of Neuropsychiatry Department of Psychiatry and Mental Health Groote Schuur Hospital Cape Town South Africa; ^3^ Alcohol, Tobacco and Other Drug Research Unit South African Medical Research Council Division of Addiction Psychiatry Department of Psychiatry and Mental Health University of Cape Town Cape Town South Africa; ^4^ Department of Psychiatry Massachusetts General Hospital/Harvard Medical School Boston MA USA; ^5^ Department of Psychology University of Miami Miami FL USA

**Keywords:** HIV, substance use, antiretroviral therapy adherence, global mental health, implementation science, South Africa

## Abstract

**Introduction:**

South Africa (SA) has the highest number of people living with HIV (PLWH) globally, and a significant burden of alcohol and other drug use (AOD). Although integrating AOD treatment into HIV care may improve antiretroviral therapy (ART) adherence, this is not typically routine practice in SA or other low‐resource settings. Identifying interventions that are feasible and acceptable for implementation is critical to improve HIV and AOD outcomes.

**Methods:**

A pilot randomized hybrid type 1 effectiveness‐implementation trial (*N* = 61) was conducted to evaluate the feasibility and acceptability of *Khanya*, a task‐shared, peer‐delivered behavioral intervention to improve ART adherence and reduce AOD in HIV care in SA. *Khanya* was compared to enhanced treatment as usual (ETAU), a facilitated referral to on‐site AOD treatment. Implementation outcomes, defined by Proctor’s model, included feasibility, acceptability, appropriateness and fidelity. Primary pilot effectiveness outcomes were ART adherence at post‐treatment (three months) measured via real‐time electronic adherence monitoring, and AOD measured using biomarker and self‐report assessments over six months. Data collection was conducted from August 2018 to April 2020.

**Results and discussion:**

Ninety‐one percent of participants (*n* = 56) were retained at six months. The intervention was highly feasible, acceptable, appropriate and delivered with fidelity (>90% of components delivered as intended by the peer). There was a significant treatment‐by‐time interaction for ART adherence (estimate = −0.287 [95% CI = −0.507, −0.066]), revealing a 6.4 percentage point increase in ART adherence in *Khanya*, and a 22.3 percentage point decline in ETAU. Both groups evidenced significant reductions in alcohol use measured using phosphatidylethanol (PEth) (*F*(2,101) = 4.16, *p* = 0.01), significantly decreased likelihood of self‐reported moderate or severe AOD (*F*(2,104) = 7.02, *p* = 0.001), and significant declines in alcohol use quantity on the timeline follow‐back (*F*(2,102) = 21.53, *p* < 0.001). Among individuals using drugs and alcohol, there was a greater reduction in alcohol use quantity in *Khanya* compared to ETAU over six months (*F*(2,31) = 3.28, *p* = 0.05).

**Conclusions:**

Results of this pilot trial provide initial evidence of the feasibility and acceptability of the *Khanya* intervention for improving adherence in an underserved group at high risk for ongoing ART non‐adherence and HIV transmission. Implementation results suggest that peers may be a potential strategy to extend task‐sharing models for behavioral health in resource‐limited, global settings.

## 1 Introduction

South Africa (SA) is home to the largest number of people living with HIV (PLWH) globally [1]. Despite a large antiretroviral therapy (ART) programme, limited ART regimens are readily available in the public health sector [2]. ART non adherence increases the risk of developing drug resistance and treatment failure, contributing to ongoing viral transmission, morbidity and mortality [3, 4].

Alongside the HIV epidemic, alcohol and other drug use (AOD) are highly prevalent among PLWH in SA [5, 6]. Individuals with AOD are at greater risk for poor ART adherence, viral non‐suppression, and ongoing HIV transmission [7, 8, 9, 10, 11, 12, 13, 14]. Integrating AOD treatment into HIV care can improve ART adherence, yet this is not typically routine practice in SA or other low‐resource settings [15, 16, 17]. Furthermore, ART adherence interventions are rarely adapted for AOD – a missed opportunity for maximizing HIV treatment outcomes [18, 19, 20, 21, 22, 23].

We conducted a hybrid type 1 effectiveness‐implementation [24] pilot study to evaluate the feasibility, acceptability, appropriateness and fidelity of a peer‐delivered intervention (“*Khanya*”), and preliminarily examine whether *Khanya* was associated with improvements in ART adherence over three months and AOD over six months versus enhanced treatment as usual (ETAU) [25, 26].

## 2 Methods

### 2.1 Recruitment and screening

Individuals were recruited between August 2018 and October 2019 from HIV care in Khayelitsha, a community with the highest HIV prevalence in the Western Cape [27]. Inclusion criteria were as follows: (1) HIV positive and on ART; (2) 18 to 65 years old; (3) at least moderate AOD on the WHO Alcohol, Smoking, and Substance Involvement Screening Test (WHO‐ASSIST [28]); (4) ART non‐adherence in the past three months, defined by either: (a) missing a pharmacy refill; (b) reinitiating first‐line treatment or being on second‐line treatment or (c) having unsuppressed viral load (≥400 copies/mL). Exclusion criteria were as follows: (1) high‐risk opiate or alcohol use warranting medical management; (2) untreated major mental illness; (3) inability to provide informed consent or speak English or isiXhosa; (4) third‐trimester pregnancy or (5) currently enrolled in AOD treatment. Eligible and interested participants completed informed consent and baseline assessments. Participants were given a Wisepill device [29] to monitor ART adherence over two weeks. At two‐weeks post‐baseline, participants were randomly assigned in parallel (1:1) to ETAU or *Khanya* using Research Electronic Data Capture (REDCap). Participants were assessed by a trained, blinded assessor at three‐ and six‐month post‐baseline. Participants received a 150ZAR (approximately $10 USD) grocery voucher for completing study assessments. All procedures were approved by the University of Cape Town Health Sciences Faculty Human Research Ethics Committee (HREC 187/2018). Magidson *et al*.[30] includes full protocol details.

### 2.2 Allocation groups

#### 2.2.1 Khanya

Khanya is a six‐session peer‐delivered behavioral intervention that integrates several evidence‐based intervention components [31, 32, 33, 34] – behavioral activation, problem solving, motivational interviewing and mindfulness‐based relapse prevention – adapted during formative work preceding this trial [35, 36]. The intervention aims to support increased ART adherence and individualized goal setting for AOD reduction by teaching evidence‐based behavioral skills (i.e. behavioral monitoring, activity scheduling, mindfulness practice, relapse prevention) to support the attainment of these goals. Home practice is assigned between sessions to reinforce skills. Real‐time electronic adherence monitoring is discussed in session in relation to skill practice to address barriers to adherence and relapse prevention. Participants are offered up to six optional booster sessions to further reinforce skills. Participants were not compensated for intervention sessions but travel costs were reimbursed. Sessions lasted approximately 60 minutes. The interventionist was a peer – an individual with lived AOD experience – paid full‐time as part of the research team, trained and supervised by clinical psychologists. Intervention, supervision and training details are provided elsewhere [30, 37].

#### 2.2.2 Enhanced treatment as usual

TAU for individuals with AOD in this context is a referral letter to Matrix [38, 39], an evidence‐based, co‐located 16‐week AOD programme that includes an initial screening and brief intervention session [25, 26]. We enhanced TAU by discussing the referral to Matrix, offering to accompany participants to the intake if they wished, and following up on referral uptake at subsequent visits.

### 2.3 Measures

Implementation outcomes were guided by Proctor’s model [40], including feasibility, acceptability, appropriateness and fidelity. *Feasibility* and *acceptability* were assessed based on uptake (percentage who initiated the intervention and session attendance respectively) and a validated quantitative measure was used for assessing implementation outcomes in Low‐and‐middle‐income countries (LMICs) [41], including feasibility, acceptability and appropriateness (ratings on a four‐point scale: 0 = “not at all”; 3 = “a lot”). *Fidelity*. A randomly selected 20% of *Khanya* sessions were rated by the interventionist and an independent coder (trained in fidelity monitoring and not involved in this study) following best practices and other studies examining fidelity of task‐shared interventions [42]. A 15 to 19 item (depending on session) checklist of core session components was developed a priori. The independent coder also rated common factors (i.e. verbal communication, self‐disclosure, normalization, empathy) using the ENhancing Assessment of Common Therapeutic factors (ENACT; 1 to 3 rating scale) [43].

ART adherence was assessed from baseline (past two weeks) through post‐treatment (approximately three months) using Wisepill, a real‐time adherence monitoring device [29]. Adherence was measured as the percentage of days’ adherent from baseline to the week prior to post‐treatment. Observations, where the device battery was dysfunctional, were excluded.

AOD was assessed using biomarker and self‐report at baseline, three months and six months. *Phosphatidylethanol (PEth)* testing was conducted from dried blood spots (≥50 ng/mL reflects unhealthy drinking up to 21 days [44]). *Urinalysis* assessed cocaine, marijuana, amphetamines, opiates, phencyclidine and alcohol (<80 hours; 300 ng/mL; [45, 46]), and methaqualone (Mandrax), a local sedative. The *WHO‐ASSIST* assessed past three‐month self‐reported AOD [28] using defined risk categories (*alcohol*: ≥27 high risk; 11 to 26 moderate; 0 to 10 low; *drugs*: ≥27 high risk; 4 to 26 moderate; 0 to 3 low). The *Timeline Follow‐Back (TLFB)* [47], a calendar‐aided assessment of AOD, assessed the quantity of alcohol use in the past two weeks and the percentage of days used (any substance). Recall was aided by the use of empty, locally recognizable alcohol containers.

Viral load (exploratory) was extracted from medical records (within three months before baseline, 30 days of follow‐ups) or drawn and tested by the National Health Laboratory Service when unavailable. Viral suppression was defined as <400 copies/mL per local clinic standards.

### 2.4 Data analytic plan

This pilot study aimed to establish the feasibility and acceptability of *Khanya*, examined using descriptive statistics (means and standard deviations) of implementation outcomes. The study sample size followed recommendations for pilot studies [48, 49]. Effectiveness outcomes were examined using multilevel modelling to account for repeated measurements [50]. Time was treated categorically to capture differing rates of change between time points, and all models included a random intercept. Analyses used an intent‐to‐treat framework [51], including all available data. Missing data were treated as missing at random. All analyses were run using SAS version 9.4 [52]. Presented models were adjusted for baseline age and gender, determined a priori [30], and observed baseline differences in theoretically relevant factors (relationship status, viral suppression and substance use severity). As sensitivity analyses, models were re‐run without covariates and with age and gender only. Models were also run with time treated continuously and a random slope. The pattern of results for sensitivity analyses did not differ. We also examined all results separately for a subsample who also used drugs in the past three months (*n* = 21). See supplemental materials for the more detailed data analytic plan.

## 3 Results and Discussion

### 3.1 Participants

**Figure 1:**
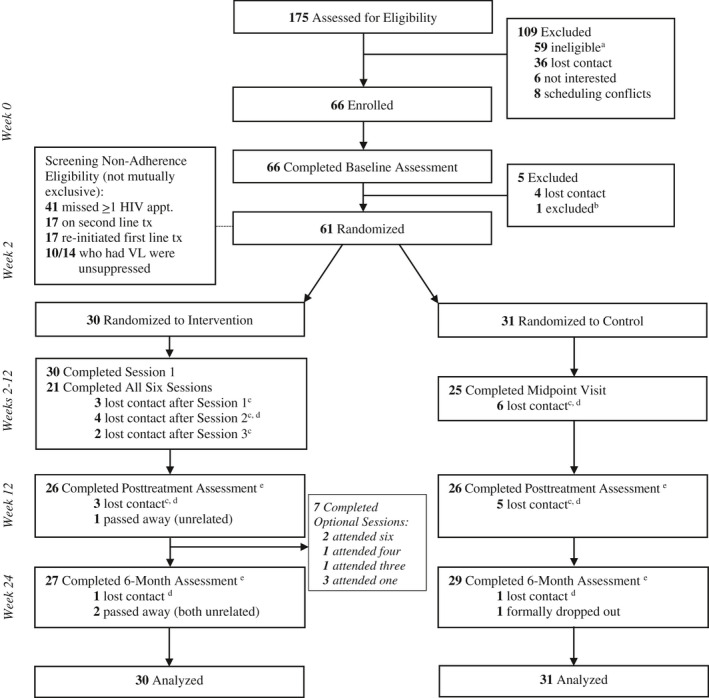
Consort diagram. ^a^WHO‐ASSIST too low only (n = 54); not struggling with adherence only (n = 1); WHO‐ASSIST too low and not struggling with adherence (n = 1); undertreated major mental illness (n = 2); incomplete screening (n = 1). ^b^1 participant excluded pre‐randomization for severe alcohol dependence (medical management of withdrawal symptoms needed). ^c^Participants uncontactable for specific event, but attended later events. ^d^1 participant was uncontactable for remainder of study. ^e^Due to staffing resource restraints, only 36/52 assessments were blinded at post‐treatment (69%), and only 19/56 assessments were blinded at six‐month follow‐up (34%).

A total of *N* = 61 participants were enrolled and randomized; see Figure 1 for consort diagram. The sample was largely Black African and 54% female. Individuals were ART adherent 51.4% of days over two weeks at baseline, over 80% had unhealthy drinking, and approximately one‐third had current drug use. Table 1 includes baseline characteristics, and Table 2 includes all outcomes by treatment group by time point.

**Table 1:** Baseline demographic and clinical characteristics of sample by treatment group

Characteristic	Total sample (*N* = 61)	Khanya (*n* = 30)	ETAU (*n* = 31)
Age, *M* (SD)	37.00 (9.63)	39.80 (10.47)	34.29 (7.99)
% Female (n)	54.1 (33)	43.3 (13)	64.5 (20)
% Graduated high school or above (n)	23.0 (14)	26.7 (8)	19.4 (6)
% Casual or full‐time employment (n)	21.3 (13)	13.3 (4)	29.0 (9)
% Married or common‐law (n)	26.2 (16)	10.0 (3)	41.9 (13)
HIV characteristics			
Years since HIV diagnosis, *M* (SD)	6.27 (4.87)	7.14 (5.97)	5.43 (3.39)
% Suppressed viral load (<400 copies/mL) (n)	63.9 (39)	50.0 (15)	77.4 (24)
CD4 count	376 (253)	330 (210)	420 (285)
% Days adherent via Wisepill over two weeks, *M* (SD)^a^	51.4 (30.7)	53.6 (32.1)	49.3 (29.7)
% On second‐line (n)^b^	26.7 (16)	24.1 (7)	29.0 (9)
Substance use characteristics			
% Positive alcohol urine test (n)	88.5 (54)	86.7 (26)	90.3 (28)
% PEth unhealthy drinking (n)	83.6 (51)	90.0 (27)	77.4 (24)
WHO‐ASSIST alcohol score, *M* (SD)^c^	25.66 (6.88)	25.93 (6.70)	25.39 (7.16)
% Days consumed any substance on TLFB, *M* (SD)	34.1 (23.5)	38.8 (24.6)	29.5 (21.7)
% Days consumed alcohol on TLFB, *M* (SD)	30.3 (21.0)	37.1 (22.9)	23.7 (16.9)
Average number of drinks on days drinking on TLFB, *M* (SD)	7.60 (4.75)	7.17 (3.79)	8.03 (5.59)^d^
% Positive (any) drug urine test (n)	31.2 (19)	40.0 (12)	22.6 (7)
WHO‐ASSIST (any) drug score, *M* (SD)^e^	13.10 (9.47)	9.25 (6.34)	15.67 (10.84)
% Days consumed other drugs on TLFB, *M* (SD)^f^	39.8 (26.0)	47.6 (35.9)	33.9 (19.7)

^a^ Data from randomization visit

^b^ n = 29 for Khanya arm

^c^ data from screening visit

^d^ n = 30

^e^ score for those who reported using substance in past 3 months (N = 11 WHO ASSIST scores from 10 participants, n = 4 Khanya arm, n = 7 ETAU arm)

^f^ Cannabis was the only substance reported at this assessment; n = 3 for Khanya arm, n = 4 or ETAU arm. TFLB, Timeline Follow‐Back; SD, Standard deviation

**Table 2:** Sample means and percentages for primary outcomes at assessment time points by treatment group

Outcome measure	Khanya			ETAU		
	Baseline (*N* = 30)	Three‐month (*N* = 26)	Six‐month (*N* = 27)	Baseline (*N* = 31)	Three‐month (*N* = 26)	Six‐month (*N* = 29)
% Days adherent via Wisepill, *M* *(SD)*	53.6 (32.1)^a^	60.0 (37.1)^b^	–	49.3 (29.8)	28.2 (32.1)^c^	–
% (n) suppressed viral load, <400 copies/mL	50.0 (15)	65.4 (17)	59.3 (16)	77.4 (24)	73.1 (19)	75.9 (22)
PEth score, *M* *(SD)*	686.0 (639.9)	484.2 (398.7)	538.4 (554.4)^d^	456.1 (530.8)	414.7 (389.6)^d^	386.1 (392.6)
% Positive alcohol or drug urine test *(n)*	90.0 (27)	96.2 (25)	83.3 (20)	93.6 (29)	88.5 (23)	82.8 (24)
% Moderate or high risk on WHO‐ASSIST	100 (30)	88.5 (23)	88.9 (24)	100 (31)	96.2 (25)	75.9 (22)
% Days consumed any substance on TLFB, *M* *(SD)*	38.8 (24.6)	29.1 (24.6)	30.7 (25.9)	29.5 (21.7)	24.5 (20.2)	25.1 (23.2)
Average number of drinks on days drinking on TLFB, *M* *(SD)*	7.17 (3.79)	5.36 (3.84)	4.61 (3.29)	8.03 (5.59)^e^	5.47 (3.77)^d^	4.96 (4.25)

^a^ *n* = 29

^b^ *n* = 28

^c^ *n* = 27

^d^ *n* = 25

^e^ *n* = 30. SD, standard deviation; TLFB, Timeline Follow‐Back

### 3.2 Outcomes

#### 3.2.1 Implementation outcomes

Although AOD treatment utilization is typically low in SA [53], treatment uptake was high in this sample. Of the participants randomized to *Khanya*, 100% initiated the intervention, and 70% attended all six sessions (*M* = 4.77; *SD* = 1.96); 88% of *Khanya* participants reported satisfaction with the number of treatment sessions. Feasibility, acceptability and appropriateness of *Khanya* were rated very highly (feasibility: *M* = 2.98; *SD* = 0.18; acceptability: *M* = 2.98; *SD* = 0.04; appropriateness: *M* = 2.94, *SD* = 0.09). For ETAU, 80.6% (*n* = 25) attended the Matrix referral, of whom 68% (*n* = 17) attended only one session (range 0 to 11). Interventionist self‐reported fidelity was 96.5% (S*D* = 7.2) for *Khanya*; average independent rater fidelity was 91.7% (*SD* = 13.3). Peer therapeutic common factors, such as warmth and non‐judgement, were rated highly using ENACT (*M* = 2.69; *SD* = 0.28).

#### 3.2.2 Effectiveness outcomes

Overall model results for all effectiveness outcomes are presented in Table 3. Drug subsample results for all outcomes are in Tables S1‐S6.

**Table 3:** Overview of all model results by time and time by treatment interaction

	Time only (effect of Khanya on outcome)				Time by Treatment (effect of ETAU on outcome)			
Effect	Estimate (SE) or DF	95% CI	*F* or *t*	*p*	Estimate (SE) or DF	95% CI	*F* or *t*	*p*
Wisepill adherence	–	–	–	–	–	–	–	–
PT	0.064 (0.078)	[−0.092, 0.220]	0.82	0.41	−0.287 (0.110)	[−0.507, −0.066]	−2.61	0.01
FU	–	–	–	–	–	–	–	–
PEth	2, 101	–	4.16	0.01	2, 101	–	0.97	0.38
PT	−227 (80)	[−385, −69]	−2.85	0.005	157 (113)	[−67, 382]	1.39	0.16
FU	−169 (81)	[−329, −9]	−2.09	0.03	84 (111)	[−136, 304]	0.76	0.44
Alcohol/drug urine test	2, 101	–	1.67	0.19	2, 101	–	0.59	0.55
PT	−1.14 (1.36)	[−3.85, 1.56]	−0.84	0.40	1.95 (1.79)	[−1.61, 5.50]	1.09	0.27
FU	0.80 (1.04)	[−1.26, 2.87]	0.77	0.44	0.71 (1.49)	[−2.24, 3.67]	0.48	0.63
Categorical ASSIST	2, 104	–	7.02	0.001	2, 104	–	0.10	0.90
PT	−1.35 (0.67)	[−2.67, −0.03]	−2.03	0.04	0.25 (0.89)	[−1.51, 2.00]	0.28	0.77
FU	−1.68 (0.66)	[−2.98, −0.37]	−2.55	0.01	−0.14 (0.87)	[−1.85, 1.58]	−0.16	0.87
TLFB average drinks	2, 102	–	21.53	<0.001	2, 102	–	0.05	0.94
PT	−0.32 (0.11)	[−0.54, −0.10]	−2.91	0.004	−0.05 (0.16)	[−0.36, 0.26]	−0.32	0.74
FU	−0.46 (0.11)	[−0.69, −0.24]	−4.07	<0.001	−0.03 (0.16)	[−0.34, 0.28]	−0.18	0.85
Viral suppression (<400)	2, 104	–	0.27	0.76	2, 104	–	0.72	0.49
PT	1.01 (0.77)	[−0.52, 2.53]	1.31	0.19	−1.31 (1.13)	[−3.56, 0.93]	−1.16	0.24
FU	0.81 (0.75)	[−0.69, 2.30]	1.07	0.28	−0.89 (1.10)	[−3.08, 1.29]	−0.81	0.41

##### ART adherence

There was a significant treatment‐by‐time interaction (estimate = −0.287 [95% CI = −0.507, −0.066]), such that ETAU’s pre‐post treatment change in days’ adherent was 28 percentage points lower than *Khanya*. Average adherence increased 6.4 percentage points in *Khanya*, whereas adherence declined by 22.3 percentage points in ETAU (see Figure S1).

Although the study was focused on implementation, the fact that we saw large effects on behavioral adherence in this small sample is noteworthy. Improving ART adherence among individuals with AOD is a known challenge [15]. Interventions to enhance adherence are critical for improving individual HIV outcomes and supporting treatment as prevention.

##### Alcohol and other drug use

###### Biomarker

There was a significant main effect of time in the model predicting PEth (*F*(2,101) = 4.16, *p* = 0.01) and a non‐significant treatment‐by‐time interaction (*F*(2,101) = 0.97, *p* = 0.38), indicating both groups demonstrated reductions in PEth. In the model predicting negative urinalysis results for drug use or past three‐day alcohol use, the time (*F*(2,101) = 1.67, *p* = 0.19) and treatment‐by‐time interaction were not significant (*F*(2,101) = 0.59, *p* = 0.55).

###### WHO alcohol, smoking, and substance involvement screening test

There was a significant main effect of time in the model predicting the likelihood of moderate or high‐risk WHO‐ASSIST category (*F*(2,104) = 7.02, *p* = 0.001), but not the treatment‐by‐time interaction (*F*(2,104) = 0.10, *p* = 0.90). The probability of being in the high‐risk category reduced 30 percentage points at three months and 40 percentage points at six months.

###### Timeline follow‐back

There was a significant effect of time in the model predicting the average number of drinks consumed for *Khanya* (*F*(2,102) = 21.53, *p* < 0.001). Participants in *Khanya* consumed on average 5.3 and 4.6 drinks at the subsequent time points, compared to 7.3 at baseline. However, the treatment‐by‐time interaction was not significant, indicating similar changes across groups over time. For the subsample who also used drugs, there was a marginally significant treatment‐by‐time interaction (*F*(2,31) = 3.28, *p* = 0.05); *Khanya* had a greater reduction in the number of drinks at six months compared to ETAU (see Figure S2). Given the *Khanya* intervention did not require abstinence, it can be understood that individuals reduced amounts of alcohol consumed rather than abstinence.

##### Viral load (exploratory)

There was no treatment effect on viral load (*F*(2,104) = 0.72, *p* = 0.49). However, we were not adequately powered in this pilot study to detect changes in viral load over a relatively short follow‐up period [54, 55], especially since 63.9% of the sample was suppressed at baseline. However, there was a significant relationship between viral suppression and adherence; individuals with higher adherence at post‐treatment were more likely to be virally suppressed (*t* = 2.31; *p* = 0.02). Results are consistent with other behavioral intervention trials with a primary focus on ART adherence that did not demonstrate treatment effects on viral load [56, 57, 58].

## 4 Conclusions

This pilot trial provides initial evidence of the feasibility and acceptability of the peer‐delivered *Khanya* intervention for improving adherence alongside AOD in South African HIV care. Peers offer a potential solution to known implementation barriers of task‐sharing behavioral interventions with CHWs, including high caseloads and other clinical demands [35, 59]. Peers bring with them lived experience, which can foster connection with patients and potentially reduce HIV and AOD stigma [60, 61]. Initial results regarding the implementation success of *Khanya* and ART adherence improvements are promising. Results suggest that engagement in AOD treatment alone without integrated adherence support may not be sufficient to improve ART adherence; however, a larger trial is needed to evaluate longer term effectiveness outcomes, including viral suppression, and to consider a stepped care approach to efficiently allocate resources to support individuals most in need of intensive intervention.

Strengths of this trial included a rigorous comparison condition, prioritization of individuals most in need of intervention – with both AOD and ART non‐adherence – use of a hybrid effectiveness‐implementation design, and high retention rates. Primary limitations relate to this being a pilot trial, including small sample size and relatively short follow‐up. As a pilot study, we were not powered to detect differences in viral load – an exploratory outcome. Furthermore, our urinalysis assessment of substance use included detection of alcohol use in the past three days; based on the alcohol use severity in this population and *Khanya*’s focus on reduction of use and harms rather than abstinence, we also had limited power to detect changes in urine‐verified abstinence. Finally, although real‐time electronic adherence monitoring is a strength, it has its limitations; Wisepill can act as an intervention in itself, and non‐use of Wisepill may be conflated with ART non‐adherence – although we limited our assessment to three months to minimize potential non‐use over time [29]. Despite these limitations, this pilot study contributes new knowledge about incorporating peers as AOD interventionists within HIV care in SA. As SA Department of Health refines its strategy for integration of behavioral health services into primary care, findings may have important implications for the feasibility and acceptability of incorporating peers into task‐shared services for improving ART adherence among PLWH with AOD.

## Competing interests

Dr. Safren receives royalties from Oxford University Press, Guilford Publications, and Springer/Humana Press for books related to cognitive behavioral therapy. All other authors declare no conflicts of interest.

## Authors’ contributions

JFM conceptualized the idea and secured funding for the project with JAJ, BM and SAS. JM led all aspects of the study and wrote the first draft of the manuscript. JAJ, BM, COC and SAS provided guidance throughout the study and provided a critical review and edits of the manuscript. JMB conducted the analyses, oversaw data management and provided edits to the manuscript. LSA provided oversight of study operations, contributed to idea conceptualization and provided a critical review of the manuscript. KSR developed the protocol with JFM and SM and led all aspects of study start up, operations, data oversight and regulatory compliance, along with SM, and contributed to manuscript writing. ALR oversaw study operations, procedures for biomarker specimen testing and analysis along with SM, and contributed to manuscript writing. All authors approve of the final manuscript to be submitted for publication.

## Supporting information


**Figure S1**. Model‐implied Wisepill adherence at baseline and post‐treatment (three‐months) for ETAU and Khanya intervention groups
**Figure S2**. Model‐implied alcohol use quantity at baseline, post‐treatment, and follow‐up time points for ETAU and intervention groups among the subsample who used both drugs and alcohol (*n* = 21)

**Table S1**. Linear model predicting wisepill adherence
**Table S2**. Linear model predicting continuous PEth and categorical model predicting dichotomous urine
**Table S3**. Cumulative logit model predicting moderate and high risk categories of WHO‐ASSIST
**Table S4**. Count model predicting average number of drinks consumed on days drinking on the timeline followback
**Table S5**. Linear model predicting percentage days used any substance on the timeline followback
**Table S6**. Categorical model predicting binary viral load suppression

## References

[1] Statistics South Africa. 2020 Mid‐year population estimates [Internet]. 2020. [cited 2020 Aug 24]. Available from: http://www.statssa.gov.za/?p=13453

[2] Moorhouse M , Maartens G , Venter WDF , Moosa M‐Y , Steegen K , Jamaloodien K , et al. Third‐line antiretroviral therapy program in the south african public sector: cohort description and virological outcomes. JAIDS J Acquir Immune Defic Syndr [Internet]. 2019;80(1): [cited 2020 Aug 24]. Available from: https://journals.lww.com/jaids/Fulltext/2019/01010/Third_Line_Antiretroviral_Therapy_Program_in_the.13.aspx 10.1097/QAI.0000000000001883 PMC6319697 30334876

[3] Nachega JB , Marconi VC , van Zyl GU , Gardner EM , Preiser W , Hong SY , et al. HIV treatment adherence, drug resistance, virologic failure: evolving concepts. Infect Disord Drug Targets. 2011;11(2):167–74. 21406048 10.2174/187152611795589663 PMC5072419

[4] Marconi VC , Sunpath H , Lu Z , Gordon M , Koranteng‐Apeagyei K , Hampton J , et al. Prevalence of HIV‐1 drug resistance after failure of a first highly active antiretroviral therapy regimen in KwaZulu Natal, South Africa. Clin Infect Dis. 2008;46(10):1589–97. 18419495 10.1086/587109 PMC2692213

[5] Viet Cuong P , Casswell S , Parker K , Callinan S , Chaiyasong S , Kazantseva E , et al. Cross‐country comparison of proportion of alcohol consumed in harmful drinking occasions using the International Alcohol Control Study. Drug Alcohol Rev. 2018;37 Suppl 2:S45–52. 29441632 10.1111/dar.12665 PMC6120468

[6] Scheibe AP , Gloeck NR , Shelly S , Marcus TS , Hugo J . The prevalence and characteristics of moderate‐ to high‐risk regulated and unregulated substance use among patients admitted to four public hospitals in Tshwane, South Africa. S Afr Med J. 2019;109(12) [Internet]. [cited 2020 Aug 24]. Available from: http://www.samj.org.za/index.php/samj/article/view/12777 10.7196/SAMJ.2019.v109i12.13870 31865961

[7] Parry CD , Londani M , Shuper PA , Myers B , Kekwaletswe CT , Nkosi S , et al. Characteristics and drinking behaviour of patients on antiretroviral therapy who drink and attend HIV clinics in Tshwane, South Africa: implications for intervention. S Afr Med J. 2019;109(10):784–91. 31635577 10.7196/SAMJ.2019.v109i10.13586

[8] Myers B. , Lombard C. , Joska J.A. , Abdullah F. , Naledi T. , Lund C. , et al. Associations Between Patterns of Alcohol Use and Viral Load Suppression Amongst Women Living with HIV in South Africa. AIDS Behav. (2021). 10.1007/s10461-021-03263-3 PMC8560660 33876383

[9] Cook RL , Sereika SM , Hunt SC , Woodward WC , Erlen JA , Conigliaro J . Problem drinking and medication adherence among persons with HIV infection. J Gen Intern Med. 2001;16(2):83–88. 11251758 10.1111/j.1525-1497.2001.00122.x PMC1495171

[10] Kader R , Seedat S , Govender R , Koch JR , Parry CD . Hazardous and harmful use of alcohol and/or other drugs and health status among South African patients attending HIV clinics. AIDS Behav. 2014;18(3):525–34. 23921585 10.1007/s10461-013-0587-9

[11] Kalichman SC , Simbayi LC , Kaufman M , Cain D , Jooste S . Alcohol use and sexual risks for HIV/AIDS in sub‐Saharan Africa: systematic review of empirical findings. Prev Sci. 2007;8(2):141–51. 17265194 10.1007/s11121-006-0061-2

[12] Patterson TL , Semple SJ , Zians JK , Strathdee SA . Methamphetamine‐using HIV‐positive men who have sex with men: correlates of polydrug use. J Urban Health. 2005;82(1):i120–26. 15738313 10.1093/jurban/jti031 PMC3456168

[13] Kekwaletswe CT , Jordaan E , Nkosi S , Morojele NK . Social support and the mediating roles of alcohol Use and adherence self‐efficacy on Antiretroviral Therapy (ART) adherence among ART recipients in Gauteng, South Africa. AIDS Behav. 2017;21(7):1846–56. 27837424 10.1007/s10461-016-1595-3

[14] Velloza J , Kemp CG , Aunon FM , Ramaiya MK , Creegan E , Simoni JM . Alcohol use and antiretroviral therapy non‐adherence among adults living with HIV/AIDS in sub‐Saharan Africa: a systematic review and meta‐analysis. AIDS Behav. 2020;24(6):1727–42. 31673913 10.1007/s10461-019-02716-0 PMC7190427

[15] Williams EC , McGinnis KA , Rubinsky AD , Matson TE , Bobb JF , Lapham GT , et al. Alcohol use and antiretroviral adherence among patients living with HIV: is change in alcohol use associated with change in adherence? AIDS Behav. 2021;25(1):203–14. 32617778 10.1007/s10461-020-02950-x PMC7775874

[16] Parsons JT , Golub SA , Rosof E , Holder C . Motivational interviewing and cognitive‐behavioral intervention to improve HIV medication adherence among hazardous drinkers. J Acquir Immune Defic Syndr. 2007;46(4):443–50. 18077833 10.1097/qai.0b013e318158a461 PMC2666542

[17] Parsons JT , John SA , Millar BM , Starks TJ . Testing the efficacy of combined motivational interviewing and cognitive behavioral skills training to reduce methamphetamine use and improve HIV medication adherence among HIV‐positive gay and bisexual Men. AIDS Behav. 2018;22(8):2674–86. 29536284 10.1007/s10461-018-2086-5 PMC6051905

[18] Haldane V , Cervero‐Liceras F , Chuah FL , Ong SE , Murphy G , Sigfrid L , et al. Integrating HIV and substance use services: a systematic review. J Int AIDS Soc [Internet]. 2017;20(1). 21585 [cited 2020 Aug 24]. Available from: https://www.ncbi.nlm.nih.gov/pmc/articles/PMC5515016/ 10.7448/IAS.20.1.21585 PMC5515016 28692211

[19] Jenkins RA . Getting to zero: we can’t do it without addressing substance use. AIDS Educ Prev. 2018;30(3):225–31. 29969312 10.1521/aeap.2018.30.3.225

[20] Dale SK , Traeger L , O’Cleirigh C , Bedoya CA , Pinkston M , Wilner JG , et al. Baseline substance use interferes with maintenance of HIV medication adherence skills. AIDS Patient Care and STDs. 2016;30(5):215–20. 27158849 10.1089/apc.2015.0340 PMC4870605

[21] Myers B , Sorsdahl K , Morojele NK , Kekwaletswe C , Shuper PA , Parry CDH . “In this thing I have everything I need”: perceived acceptability of a brief alcohol‐focused intervention for people living with HIV. AIDS Care. 2017;29(2):209–13. 27435957 10.1080/09540121.2016.1211242

[22] Kekwaletswe CT , Morojele NK . Alcohol use, antiretroviral therapy adherence, and preferences regarding an alcohol‐focused adherence intervention in patients with human immunodeficiency virus. Patient Prefer Adherence. 2014;31(8):401–13. 10.2147/PPA.S55547 PMC3976236 24729688

[23] Myers B , Parry CDH , Morojele NK , Nkosi S , Shuper PA , Kekwaletswe CT , et al. “Moving forward with life”: acceptability of a brief alcohol reduction intervention for people receiving antiretroviral therapy in South Africa. Int J Environ Res Public Health. 2020;17:5706. 10.3390/ijerph17165706 PMC7459709 32784613

[24] Curran GM , Bauer M , Mittman B , Pyne JM , Stetler C . Effectiveness‐implementation hybrid designs: combining elements of clinical effectiveness and implementation research to enhance public health impact. Med Care. 2012;50(3):217–26. 22310560 10.1097/MLR.0b013e3182408812 PMC3731143

[25] Gouse H , Magidson JF , Burnhams W , Remmert JE , Myers B , Joska JA , et al. Implementation of cognitive‐behavioral substance abuse treatment in sub‐Saharan Africa: treatment engagement and abstinence at treatment exit. PLoS One [Internet]. 2016; Jan 27 [cited 2019 Nov 22];11(1). Available from: https://www.ncbi.nlm.nih.gov/pmc/articles/PMC4729488/ 10.1371/journal.pone.0147900 PMC4729488 26816208

[26] Magidson JF , Gouse H , Burnhams W , Wu CYY , Myers B , Joska JA , et al. Beyond methamphetamine: documenting the implementation of the Matrix model of substance use treatment for opioid users in a South African setting. Addict Behav. 2017;66:132–7. 27940387 10.1016/j.addbeh.2016.11.014 PMC5221678

[27] Western Cape Provincial AIDS Council . Annual Progress Report 2014/15 [Internet]. South Africa: Western Cape Government; 2016 Mar. (Provincial Strategic Plan 2012‐2016). [cited 2020 Aug 24]. Available from: http://sanac.org.za/wp‐content/uploads/2016/04/WC_PSP‐ANNUAL‐PROGRESS‐REPORT‐Final.pdf

[28] WHO Assist Working Group . The alcohol, smoking and substance involvement screening test (ASSIST): development, reliability and feasibility. Addiction. 2002;97(9):1183–94. 12199834 10.1046/j.1360-0443.2002.00185.x

[29] Haberer JE , Kahane J , Kigozi I , Emenyonu N , Hunt P , Martin J , et al. Real‐time adherence monitoring for HIV antiretroviral therapy. AIDS Behav. 2010;14(6):1340–6. 20809380 10.1007/s10461-010-9799-4 PMC2974938

[30] Magidson JF , Joska JA , Myers B , Belus JM , Regenauer KS , Andersen LS , et al. Project Khanya: a randomized, hybrid effectiveness‐implementation trial of a peer‐delivered behavioral intervention for ART adherence and substance use in Cape Town, South Africa. Implement Sci Commun. 2020;1(23). 10.1186/s43058-020-00004-w PMC7326344 32607502

[31] Safren S , Otto M , Worth J . Life‐steps: applying cognitive behavioral therapy to HIV medication adherence. Sci Direct Cognit Behav Pract. 1999;6(4):332–41.

[32] Daughters SB , Magidson JF , Anand D , Seitz‐Brown CJ , Chen Y , Baker S . The effect of a behavioral activation treatment for substance use on post‐treatment abstinence: a randomized controlled trial. Addiction. 2018;113(3):535–44. 28963853 10.1111/add.14049 PMC5807178

[33] Bowen S , Witkiewitz K , Clifasefi SL , Grow J , Chawla N , Hsu SH , et al. Relative efficacy of mindfulness‐based relapse prevention, standard relapse prevention, and treatment as usual for substance use disorders: a randomized clinical trial. JAMA Psychiatry. 2014;71(5):547–56. 24647726 10.1001/jamapsychiatry.2013.4546 PMC4489711

[34] Magill M , Apodaca TR , Borsari B , Gaume J , Hoadley A , Gordon REF , et al. A meta‐analysis of motivational interviewing process: Technical, relational, and conditional process models of change. J Consult Clin Psychol. 2018;86(2):140–57. 29265832 10.1037/ccp0000250 PMC5958907

[35] Magidson JF , Joska JA , Regenauer KS , Satinsky E , Andersen LS , Seitz‐Brown CJ , et al. “Someone who is in this thing that I am suffering from”: the role of peers and other facilitators for task sharing substance use treatment in South African HIV care. Int J Drug Policy. 2019;70:61–9. 31082664 10.1016/j.drugpo.2018.11.004 PMC6679990

[36] Magidson JF , Andersen LS , Satinsky EN , Myers B , Kagee A , Anvari M , et al. “Too much boredom isn’t a good thing”: adapting behavioral activation for substance use in a resource‐limited South African HIV care setting. Psychotherapy. 2020;57(1):107–18. 31670529 10.1037/pst0000257 PMC7069775

[37] Belus JM , Rose AL , Andersen LS , Ciya N , Joska JA , Myers B , et al. Adapting a behavioral intervention for alcohol use and HIV medication adherence for lay counselor delivery in Cape Town, South Africa: A case series. Cognitive and Behavioral Practice. 2020. 10.1016/j.cbpra.2020.10.003 PMC9512118 36171964

[38] Rawson RA , Shoptaw SJ , Obert JL , McCann MJ , Hasson AL , Marinelli‐Casey PJ , et al. An intensive outpatient approach for cocaine abuse treatment: the Matrix model. J Subst Abuse Treat. 1995;12(2):117–27. 7623389 10.1016/0740-5472(94)00080-b

[39] Obert JL , McCann MJ , Marinelli‐Casey P , Weiner A , Minsky S , Brethen P , et al. The matrix model of outpatient stimulant abuse treatment: history and description. J Psychoactive Drugs. 2000;32(2):157–64. 10908003 10.1080/02791072.2000.10400224

[40] Proctor E , Silmere H , Raghavan R , Hovmand P , Aarons G , Bunger A , et al. Outcomes for implementation research: conceptual distinctions, measurement challenges, and research agenda. Adminis Policy Mental Health Mental Health Serv Res. 2011;38(2):65–76. 10.1007/s10488-010-0319-7 PMC3068522 20957426

[41] Haroz EE , Bolton P , Nguyen AJ , Lee C , Bogdanov S , Bass J , et al. Measuring implementation in global mental health: validation of a pragmatic implementation science measure in eastern Ukraine using an experimental vignette design. BMC Health Serv Res. 2019;19(1):262. 31036002 10.1186/s12913-019-4097-y PMC6489318

[42] Dorsey S , Lyon AR , Pullmann MD , Jungbluth N , Berliner L , Beidas R . Behavioral rehearsal for analogue fidelity: feasibility in a state‐funded children's mental health initiative. Adm Policy Ment Health. 2017;44(3):395–404. 26966103 10.1007/s10488-016-0727-4 PMC5734939

[43] Kohrt BA , Jordans MJD , Rai S , Shrestha P , Luitel NP , Ramaiya MK , et al. Therapist competence in global mental health: development of the ENhancing Assessment of Common Therapeutic factors (ENACT) rating scale. Behav Res Ther. 2015;1(69):11–21. 10.1016/j.brat.2015.03.009 PMC4686771 25847276

[44] Magidson JF , Fatch R , Orrell C , Amanyire G , Haberer JE , Hahn JA , et al. Biomarker‐measured unhealthy alcohol use in relation to CD4 count among individuals starting ART in Sub‐Saharan Africa. AIDS Behav. 2019;23(6):1656–67. 30560484 10.1007/s10461-018-2364-2 PMC6535416

[45] Vinikoor MJ , Zyambo Z , Muyoyeta M , Chander G , Saag MS , Cropsey K . Point‐of‐care urine ethyl glucuronide testing to detect alcohol use among HIV‐hepatitis B virus coinfected adults in Zambia. AIDS Behav. 2018;22(7):2334–9. 29336004 10.1007/s10461-018-2030-8 PMC6021200

[46] Confirm Biosciences . DrugConfirm Advanced Urine Drug Test Cups in Bulk | 10 Panel Drug Test [Internet]. [cited 2020 Sep 9]. Available from: https://www.confirmbiosciences.com/products/urine‐drug‐test‐cups/drugconfirm‐advanced‐drug‐test‐cup/

[47] Sobell LC , Sobell MB . Timeline follow‐back. In: Litten RZ , Allen JP , editors. Measuring alcohol consumption. Springer; 1992. p. 41–72.

[48] Rounsaville BJ , Carroll KM , Onken LS . A stage model of behavioral therapies research: getting started and moving on from stage I. Clin Psychol Sci Pract. 2001;8(2):133–42.

[49] Freedland KE . Pilot trials in health‐related behavioral intervention research: problems, solutions, and recommendations. Health Psychol. 2020;39(10):851–62. 32614197 10.1037/hea0000946

[50] Raudenbush SW , Bryk AS . Hierarchical linear models: applications and data analysis methods, vol. 1. Thousand Oaks, CA: Sage Publications; 2002.

[51] Chakraborty H , Gu H . A mixed model approach for intent‐to‐treat analysis in longitudinal clinical trials with missing values. NC: Research Triangle Park; 2009. 30896910

[52] SAS Enterprise Miner. Cary, NC: SAS Institute Inc.; 2013.

[53] Degenhardt L , Glantz M , Evans‐Lacko S , Sadikova E , Sampson N , Thornicroft G , et al. Estimating treatment coverage for people with substance use disorders: an analysis of data from the World Mental Health Surveys. World Psychiatry. 2017;16(3):299–307. 28941090 10.1002/wps.20457 PMC5608813

[54] Sethi AK , Celentano DD , Gange SJ , Moore RD , Gallant JE . Association between adherence to antiretroviral therapy and human immunodeficiency virus drug resistance. Clin Infect Dis. 2003;37(8):1112–8. 14523777 10.1086/378301

[55] Orrell C , Cohen K , Leisegang R , Bangsberg DR , Wood R , Maartens G . Comparison of six methods to estimate adherence in an ART‐naïve cohort in a resource‐poor setting: which best predicts virological and resistance outcomes? AIDS Res Therapy. 2017;14(1):1. 10.1186/s12981-017-0138-y PMC5379739 28376815

[56] Safren SA , O'Cleirigh C , Tan JY , Raminani SR , Reilly LC , Otto MW , et al. A randomized controlled trial of cognitive behavioral therapy for adherence and depression (CBT‐AD) in HIV‐infected individuals. Health Psychol. 2009;28(1):1. 19210012 10.1037/a0012715 PMC2643364

[57] Safren SA , O'Cleirigh CM , Bullis JR , Otto MW , Stein MD , Pollack MH . Cognitive behavioral therapy for adherence and depression (CBT‐AD) in HIV‐infected injection drug users: a randomized controlled trial. J Consult Clin Psychol. 2012;80(3):404. 22545737 10.1037/a0028208 PMC3365619

[58] Safren SA , Bedoya CA , O'Cleirigh C , Biello KB , Pinkston MM , Stein MD , et al. Cognitive behavioural therapy for adherence and depression in patients with HIV: a three‐arm randomised controlled trial. Lancet HIV. 2016;3(11):e529–38. 27658881 10.1016/S2352-3018(16)30053-4 PMC5321546

[59] Musyimi CW , Mutiso VN , Ndetei DM , Unanue I , Desai D , Patel SG , et al. Mental health treatment in Kenya: task‐sharing challenges and opportunities among informal health providers. Int J Ment Health Syst. 2017;11:45. 28775764 10.1186/s13033-017-0152-4 PMC5540195

[60] Jack HE , Oller D , Kelly J , Magidson JF , Wakeman SE . Addressing substance use disorder in primary care: the role, integration, and impact of recovery coaches. Substance Abuse. 2018;39(3):307–14. 28991516 10.1080/08897077.2017.1389802

[61] Miler JA , Carver H , Foster R , Parkes T . Provision of peer support at the intersection of homelessness and problem substance use services: a systematic “state of the art” review. BMC Public Health. 2020;20(1):641. 32381086 10.1186/s12889-020-8407-4 PMC7203893

