# Supplementary material for: Project Khanya: results from a pilot randomized type 1 hybrid effectiveness‐implementation trial of a peer‐delivered behavioural intervention for ART adherence and substance use in HIV care in South Africa
Source: J Int AIDS Soc. 2021 Jun 24;24(Suppl 2):e25720. doi: 10.1002/jia2.25720 (PMC8222840; doi:10.1002/jia2.25720)
Supplement: Supplementary file 1 — Figure S1. Model‐implied Wisepill adherence at baseline and post‐treatment (three‐months) for ETAU and Khanya intervention groups Figure S2. Model‐implied alcohol use quantity at baseline, post‐treatment, and follow‐up time points for ETAU and intervention groups among the subsample who used both drugs and alcohol (n = 21) [file JIA2-24-e25720-s001.docx]

Supplemental Figures

Figure S1

*Model-implied Wisepill adherence at baseline and post-treatment (three-months) for ETAU and Khanya intervention groups*

Note. BL = baseline; PT = post-treatment (three months)

Figure S2

*Model-implied alcohol use quantity at baseline, post-treatment, and follow-up time points for ETAU and intervention groups among the subsample who used both drugs and alcohol (n=21).*

Note. BL = baseline; PT = post-treatment (three months)
