# Supplementary material for: Project Khanya: results from a pilot randomized type 1 hybrid effectiveness‐implementation trial of a peer‐delivered behavioural intervention for ART adherence and substance use in HIV care in South Africa
Source: J Int AIDS Soc. 2021 Jun 24;24(Suppl 2):e25720. doi: 10.1002/jia2.25720 (PMC8222840; doi:10.1002/jia2.25720)
Supplement: Supplementary file 2 — Table S1. Linear model predicting wisepill adherence Table S2. Linear model predicting continuous PEth and categorical model predicting dichotomous urine Table S3. Cumulative logit model predicting moderate and high risk categories of WHO‐ASSIST Table S4. Count model predicting average number of drinks consumed on days drinking on the timeline followback Table S5. Linear model predicting percentage days used any substance on the timeline followback Table S6. Categorical model predicting binary viral load suppression [file JIA2-24-e25720-s002.docx]

Supplemental Tables

Table S1

*Linear Model Predicting Wisepill Adherence*

|  | Full sample (*n*= 61) | | | | Drug subsample (*n* = 21) | | | |
| --- | --- | --- | --- | --- | --- | --- | --- | --- |
| Effect | Estimate (SE) | 95% CI | t | p | Estimate (SE) | 95% CI | t | p |
| Intercept | .593 (.110) | [.372, .815] | 5.38 | <.001 | .468 (.219) | [-.001, .938] | 2.14 | .05 |
| Women (intercept) | -.016 (.076) | [-.169, .136] | -.21 | .83 | -.122 (.190) | [-.529, .285] | -.64 | .53 |
| Age (intercept) | .006 (.004) | [-.002, .015] | 1.51 | .13 | .003 (.008) | [-.015, .021] | .38 | .70 |
| Married/living together  (intercept) | .035 (.088) | [-.142, .211] | .39 | .69 | -.056 (.210) | [-.505, .394] | -.27 | .79 |
| Virally unsuppressed  (intercept) | -.128 (.076) | [-.280, .023] | -1.70 | .09 | -.006 (.190) | [-.413, .400] | -.03 | .97 |
| TLFB % days used any  substance (intercept) | -.0003 (.002) | [-.004, .003] | -.17 | .86 | -.002 (.003) | [-.009, .005] | -.65 | .52 |
| ETAU (intercept) | -.050 (.094) | [-.239, .138] | -.54 | .59 | .232 (.206) | [-.209, .674] | 1.13 | .27 |
| Time (PT) | .064 (.078) | [-.092, .220] | .82 | .41 | .207 (.114) | [-.033, .447] | 1.82 | .08 |
| Time x treatment group (PT for ETAU) | -.287 (.110) | [-.507, -.066] | -2.61 | .01 | -.440 (.167) | [-.792, -.089] | -2.64 | .01 |

*Note.* TLFB = timeline followback. PT = post-treatment (three months). FU = follow-up (six-months).

Table S2

*Linear Model Predicting Continuous PEth and Categorical Model Predicting Dichotomous Urine*

|  | PEth results full sample (*n*= 61) | | | | PEth results drug subsample (*n* = 21) | | | | Urine results full sample (*n*= 61) | | | |
| --- | --- | --- | --- | --- | --- | --- | --- | --- | --- | --- | --- | --- |
| Effect | Estimate (SE) or DF | 95% CI | F or t | p | Estimate (SE) or DF | 95% CI | F or t | p | Estimate (SE) or DF | 95% CI | F or t | p |
| Intercept | 416 (151) | [113, 718] | 2.76 | .008 | 550 (288) | [-68, 1167] | 1.91 | .07 | -4.28 (2.27) | [-8.83, .26] | -1.89 | .06 |
| Women (intercept) | -155 (103) | [-361, 51] | -1.51 | .13 | -184 (228) | [-672, 304] | -.81 | .43 | 1.54 (1.13) | [-.73, 3.80] | 1.36 | .17 |
| Age (intercept) | 13 (6) | [1, 24] | 2.22 | .03 | 15 (10) | [-7, 38] | 1.44 | .17 | .05 (.06) | [-.07, .16] | .80 | .42 |
| Married/living together  (intercept) | 139 (122) | [-105, 383] | 1.14 | .25 | 218 (266) | [-352, 788] | .82 | .42 | 1.14 (1.18) | [-1.23, 3.51] | .96 | .34 |
| Virally unsuppressed  (intercept) | 293 (103) | [86, 500] | 2.84 | .006 | 328 (247) | [-202, 859] | 1.33 | .20 | -.71 (1.06) | [-2.84, 1.41] | -.67 | .50 |
| TLFB % days used any  substance (intercept) | 4 (2) | [-1, 8] | 1.63 | .10 | 2 (4) | [-6, 11] | .60 | .55 | -.002 (.023) | [-.048, .044] | -.09 | .92 |
| ETAU (intercept) | -57 (123) | [-304, 189] | -.47 | .64 | -421 (267) | [-994, 152] | -1.58 | .13 | -1.23 (1.45) | [-4.14, 1.68] | -.85 | .40 |
| Time^§^ | 2, 101 | -- | 4.16 | .01 | 2, 32 | -- | 1.09 | .34 | 2, 101 | -- | 1.67 | .19 |
| PT | -227 (80) | [-385, -69] | -2.85 | .005 | -337 (163) | [-668, -5] | -2.07 | .04 | -1.14 (1.36) | [-3.85, 1.56] | -.84 | .40 |
| FU | -169 (81) | [-329 -9] | -2.09 | .03 | -215 (156) | [-533, 103] | -1.38 | .17 | .80 (1.04) | [-1.26, 2.87] | .77 | .44 |
| Time x treatment group^§^ | 2, 101 | -- | .97 | .38 | 2, 32 | -- | 1.37 | .26 | 2, 101 | -- | .59 | .55 |
| PT for ETAU | 157 (113) | [-67, 382] | 1.39 | .16 | 330 (238) | [-156, 815] | 1.38 | .17 | 1.95 (1.79) | [-1.61, 5.50] | 1.09 | .27 |
| FU for ETAU | 84 (111) | [-136, 304] | .76 | .44 | 333 (229) | [-133, 799] | 1.46 | .15 | .71 (1.49) | [-2.24, 3.67] | .48 | .63 |

*Note.* TLFB = timeline followback. PT = post-treatment (three months). FU = follow-up (six months). ^§^ = F test

Table S3

*Cumulative Logit Model Predicting Moderate and High Risk Categories of WHO-ASSIST*

|  | Full sample (*n*= 61) | | | | Drug subsample (*n* = 21) | | | |
| --- | --- | --- | --- | --- | --- | --- | --- | --- |
| Effect | Estimate (SE) or DF | 95% CI | F or t | p | Estimate (SE) or DF | 95% CI | F or t | p |
| Intercept (moderate or high risk group) | 3.27 (1.00) | [1.26, 5.28] | 3.27 | .001 | 4.04 (1.46) | [.88, 7.19] | 2.76 | .01 |
| Intercept (high risk group) | -.92 (.93) | [-2.78, .94] | -.99 | .32 | -.13 (1.19) | [-2.70, 2.45] | -.11 | .91 |
| Women (intercept) | .25 (.61) | [-.97, 1.48] | .42 | .67 | -1.65 (.90) | [-3.59, .30] | -1.82 | .09 |
| Age (intercept) | -.03 (.03) | [-.09, .04] | -.79 | .43 | -.03 (.04) | [-.13, .06] | -.80 | .43 |
| Married/living together  (intercept) | .18 (.72) | [-1.26, 1.61] | .24 | .80 | 2.08 (1.17) | [-.45, 4.61] | 1.77 | .09 |
| Virally unsuppressed  (intercept) | .22 (.61) | [-1.01, 1.45] | .36 | .72 | .17 (.98) | [-1.94, 2.29] | .18 | .86 |
| TLFB % days used any  substance (intercept) | .04 (.01) | [.01, .07] | 2.78 | .007 | .03 (.02) | [-.01, .07] | 1.86 | .08 |
| ETAU (intercept) | -.49 (.82) | [-2.14, 1.16] | -.60 | .55 | -1.22 (1.25) | [-3.92, 1.49] | -.97 | .34 |
| Time^§^ | 2, 104 | -- | 7.02 | .001 | 2, 33 | -- | 2.60 | .08 |
| PT | -1.35 (.67) | [-2.67, -.03] | -2.03 | .04 | -1.63 (1.12) | [-3.90, .65] | -1.46 | .15 |
| FU | -1.68 (.66) | [-2.98, -.37] | -2.55 | .01 | -2.36 (1.11) | [-4.61, -.11] | -2.13 | .04 |
| Time x treatment group^§^ | 2, 104 | -- | .10 | .90 | 2, 33 | -- | .47 | .62 |
| PT for ETAU | .25 (.89) | [-1.51, 2.00] | .28 | .77 | 1.53 (1.58) | [-1.69, 4.75] | .96 | .34 |
| FU for ETAU | -.14 (.87) | [-1.85, 1.58] | -.16 | .87 | .92 (1.49) | [-2.13, 3.96] | .61 | .54 |
| Predicted probability (across groups) | Low risk group | Moderate risk group | High risk group |  | Low risk group | Moderate risk group | High risk group |  |
| Baseline | .009 | .364 | .627 |  | .008 | .327 | .665 |  |
| PT | .030 | .640 | .330 |  | .018 | .526 | .456 |  |
| FU | .049 | .723 | .228 |  | .050 | .722 | .228 |  |

*Note.* TLFB = timeline followback. PT = post-treatment (three months). FU = follow-up (six months). ^§^ = F test

Table S4

*Count Model Predicting Average Number of Drinks Consumed on Days Drinking on the Timeline Followback*

|  | Full sample (*n* = 61) | | | | Drug subsample (*n* = 21) | | | |
| --- | --- | --- | --- | --- | --- | --- | --- | --- |
| Effect | Estimate (SE) or DF | 95% CI | F or t | p | Estimate (SE) or DF | 95% CI | F or t | p |
| Intercept | 1.45 (.24) | [.96, 1.93] | 5.95 | <.001 | 1.47 (.38) | [.65, 2.29] | 3.83 | .001 |
| Women (intercept) | .24 (.16) | [-.10, .56] | 1.43 | .15 | -.37 (.30) | [-1.00, .27] | -1.24 | .23 |
| Age (intercept) | -.01 (.01) | [-.03, .01] | -1.47 | .14 | -.01 (.01) | [-.04, .02] | -.69 | .50 |
| Married/living together  (intercept) | .23 (.19) | [-.16, .61] | 1.19 | .23 | .29 (.34) | [-.44, 1.03] | .85 | .40 |
| Virally unsuppressed  (intercept) | .16 (.17) | [-.17, .49] | .98 | .33 | .51(.33) | [-.20, 1.21] | 1.54 | .14 |
| TLFB % days used any  substance (intercept) | .007 (.004) | [-.001, .014] | 1.84 | .07 | .006 (.005) | [-.005, .017] | 1.10 | .28 |
| ETAU (intercept) | -.03 (.18) | [-.40, .34] | -.16 | .87 | -.09 (.35) | [-.84, .65] | -.27 | .79 |
| Time^§^ | 2, 102 | -- | 21.53 | <.001 | 2, 31 | -- | 15.78 | <.001 |
| PT | -.32 (.11) | [-.54, -.10] | -2.91 | .004 | -.57 (.18) | [-.94, -.20] | -3.13 | .003 |
| FU | -.46 (.11) | [-.69, -.24] | -4.07 | <.001 | -1.07 (.21) | [-1.49, -.65] | -5.22 | <.001 |
| Time x treatment group^§^ | 2, 102 | -- | .05 | .94 | 2, 31 | -- | 3.28 | .05 |
| PT for ETAU | -.05 (.16) | [-.36, .26] | -.32 | .74 | -.23 (.33) | [-.91, .45] | -.70 | .49 |
| FU for ETAU | -.03 (.16) | [-.34, .28] | -.18 | .85 | .65 (.30) | [.03, 1.27] | 2.13 | .04 |

*Note.* TLFB = timeline followback. PT = post-treatment (three months). FU = follow-up (six months). ^§^ = F test

Table S5

*Linear Model Predicting Percentage Days Used Any Substance on the Timeline Followback*

|  | Full sample (*n*= 61) | | | | Drug subsample (*n* = 21) | | | |
| --- | --- | --- | --- | --- | --- | --- | --- | --- |
| Effect | Estimate (SE) or DF | 95% CI | F or t | p | Estimate (SE) or DF | 95% CI | F or t | p |
| Intercept | 47.2 (5.3) | [36.6, 57.8] | 8.92 | <.001 | 49.4 (10.7) | [26.7, 72.2] | 4.63 | <.001 |
| Women (intercept) | -17.0 (4.6) | [-26.3, -7.8] | -3.70 | <.001 | -25.2 (9.3) | [-45.0, -5.3] | -2.70 | .01 |
| Age (intercept) | .1 (.3) | [-.4, .6] | .28 | .77 | .2 (.5) | [-.8, 1.2] | .37 | .71 |
| Married/living together  (intercept) | -7.1 (5.4) | [-17.9, 3.8] | -1.31 | .19 | -10.9 (11.4) | [-35.1, 13.3] | -.96 | .35 |
| Virally unsuppressed  (intercept) | -.9 (4.8) | [-10.5, 8.6] | -.20 | .84 | -4.1 (10.8) | [-27.2, 19.1] | -.37 | .71 |
| ETAU (intercept) | -3.3 (6.3) | [-16.0, 9.4] | -.52 | .60 | 5.0 (13.0) | [-22.7, 32.8] | .39 | .70 |
| Time^§^ | 2, 104 |  | 2.41 | .09 | 2, 33 | -- | .57 | .57 |
| PT | -10.6 (5.1) | [-20.6, -.5] | -2.09 | .03 | 4.2 (10.5) | [-17.1, 25.5] | .40 | .68 |
| FU | -7.4 (5.0) | [-17.3, -2.5] | -1.48 | .14 | -5.7 (9.8) | [-25.6, 14.2] | -.58 | .56 |
| Time x treatment group^§^ | 2, 104 | -- | .42 | .66 | 2, 33 | -- | 1.07 | .35 |
| PT for ETAU | 6.5 (7.1) | [-7.7, 20.7] | .91 | .36 | -21.2 (15.4) | [-52.6, 10.2] | -1.38 | .17 |
| FU for ETAU | 3.3 (7.0) | [-10.5, 17.1] | .47 | .63 | -3.1 (14.7) | [-32.9, 26.8] | -.21 | .83 |

*Note.* PT = post-treatment (three-months). FU = follow-up (six-months). ^§^ = F test

Table S6

*Categorical Model Predicting Binary Viral Load Suppression*

|  | Full sample (*n*= 61) | | | | Drug subsample (*n* = 21) | | | |
| --- | --- | --- | --- | --- | --- | --- | --- | --- |
| Effect | Estimate (SE) or DF | 95% CI | t | p | Estimate (SE) or DF | 95% CI | t | p |
| Intercept | -.76 (1.12) | [-3.02, 1.49] | -.68 | .50 | -1.85 (1.75) | [-5.57, 1.87] | -1.06 | .30 |
| Women (intercept) | .81 (.84) | [-.87, 2.48] | .97 | .33 | -.01 (1.51) | [-3.23, 3.22] | -.01 | .99 |
| Age (intercept) | -.07 (.05) | [-.17, .03] | -1.48 | .14 | -.10 (.07) | [-.26, .06] | -1.37 | .19 |
| Married/living together  (intercept) | -.31 (1.03) | [-2.37, 1.74] | -.30 | .76 | .59 (1.75) | [-3.14, 4.32] | .34 | .73 |
| TLFB % days used any  substance (intercept) | .02 (.02) | [-.02, .05] | .84 | .40 | .02 (.03) | [-.04, .08] | .81 | .43 |
| ETAU (intercept) | 2.03 (1.12) | [-.22, 4.27] | 1.81 | .07 | 3.83 (2.18) | [-.80, 8.47] | 1.76 | .09 |
| Time^§^ | 2, 104 | -- | .27 | .76 | 2, 33 | -- | .36 | .70 |
| PT | 1.01 (.77) | [-.52, 2.53] | 1.31 | .19 | -.06 (1.35) | [-2.81, 2.69] | -.05 | .96 |
| FU | .81 (.75) | [-.69, 2.30] | 1.07 | .28 | -.54 (1.29) | [-3.17, 2.10] | -.41 | .68 |
| Time x treatment group^§^ | 2, 104 | -- | .72 | .49 | 2, 33 | -- | .15 | .85 |
| PT for ETAU | -1.31 (1.13) | [-3.56, .93] | -1.16 | .24 | -1.15 (2.09) | [-5.40, 3.09] | -.55 | .58 |
| FU for ETAU | -.89 (1.10) | [-3.08, 1.29] | -.81 | .41 | -.58 (2.02) | [-4.70, 3.53] | -.29 | .77 |
| *Note.* TLFB = timeline followback. PT = post-treatment (three-months). FU = follow-up (six-months). ^§^ = F test | | | | | | | | |

Supplemental data analytic plan

Percentage of days adherent to ART, percentage of days used on the TLFB, and PEth scores were modeled as continuous variables. Urinalysis and viral load were treated dichotomously. WHO-ASSIST categorical risk-level (low, moderate, or high) was modeled using a cumulative logistic model. The proportional odds assumption was visually assessed using empirical logits. Average number of drinks on the TLFB was rounded to the nearest whole number and treated as a count variable due to a high number of zeros. All analyses were run using SAS version 9.4. PROC GLIMMIX was used to model all non-continuous outcomes and percentage of days ART adherent because there were only two time points. The Laplace estimation method was used for the non-continuous outcomes, which approximates maximum likelihood and produces less biased estimates in small samples (1). Restricted information maximum likelihood (REML) was used for ART adherence. PROC MIXED was used to model all continuous outcomes using REML. All models included a random intercept. All analyses were conducted on the full sample and a subsample of individuals who used drugs in the past 3 months (*n*=21), either per self-report at screening (*n*=8) or urine toxicology at baseline (*n*=13).

References

1. SAS Institute Inc. The GLIMMIX procedure. In: SAS/STAT 151 User’s Guide. Cary, NC: SAS Institute Inc; 2018.
